# TRANSTHYRETIN-LIKE and BYPASS1-LIKE co-regulate growth and cold tolerance in *Arabidopsis*

**DOI:** 10.1186/s12870-020-02534-w

**Published:** 2020-07-14

**Authors:** Tao Chen, Wei Zhang, Gang Yang, Jia-Hui Chen, Bi-Xia Chen, Rui Sun, Hua Zhang, Li-Zhe An

**Affiliations:** 1 The Key Laboratory of Cell Activities and Stress Adaptations, Ministry of Education, School of Life Sciences, Lanzhou University, Lanzhou, 730000 People’s Republic of China; 2 School of Forestry, Beijing Forestry University, Beijing, 100083 People’s Republic of China

**Keywords:** BYPASS1-LIKE, TRANSTHYRETIN-LIKE, Plant growth, Cold tolerance, *Arabidopsis*

## Abstract

**Background:**

Cold stress inhibits normal physiological metabolism in plants, thereby seriously affecting plant development. Meanwhile, plants also actively adjust their metabolism and development to adapt to changing environments. Several cold tolerance regulators have been found to participate in the regulation of plant development. Previously, we reported that BYPASS1-LIKE (B1L), a DUF793 family protein, participates in the regulation of cold tolerance, at least partly through stabilizing C-REPEAT BINDING FACTORS (CBFs). In this study, we found that B1L interacts with TRANSTHYRETIN-LIKE (TTL) protein, which is involved in brassinosteroid (BR)-mediated plant growth and catalyses the synthesis of S-allantoin, and both proteins participate in modulating plant growth and cold tolerance.

**Results:**

The results obtained with yeast two hybrid (Y2H) and bimolecular fluorescence complementation (BiFC) assays showed that B1L directly interacted with TTL. Similar to the *ttl-1* and *ttl-2* mutants, the *b1l* mutant displayed a longer hypocotyl and greater fresh weight than wild type, whereas B1L-overexpressing lines exhibited a shorter hypocotyl and reduced fresh weight. Moreover, *ttl-1* displayed freezing tolerance to cold treatment compared with WT, whereas the *b1l* mutant and TTL-overexpressing lines were freezing-sensitive. The *b1l ttl* double mutant had a developmental phenotype and freezing tolerance that were highly similar to those of *ttl-1* compared to *b1l*, indicating that TTL is important for B1L function. Although low concentrations of brassinolide (0.1 or 1 nM) displayed similarly promoted hypocotyl elongation of WT and *b1l* under normal temperature, it showed less effect to the hypocotyl elongation of *b1l* than to that of WT under cold conditions. In addition, the *b1l* mutant also contained less amount of allantoin than Col-0.

**Conclusion:**

Our results indicate that B1L and TTL co-regulate development and cold tolerance in *Arabidopsis*, and BR and allantoin may participate in these processes through B1L and TTL.

## Background

As sessile organisms, plants adjust their growth and development to adapt to fluctuating environments throughout their life cycle. The adaptation of plants to extreme environments requires complex physiological and biochemical processes. Large numbers of proteins have been found to play important roles in modulating plant cold tolerance [1–3]. Among these proteins, several core regulators of cold tolerance, such as C-REPEAT BINDING FACTORS (CBFs) and INDUCER OF CBF EXPRESSION 1 (ICE1) [4–7], have been found to regulate diverse developmental processes [8–15].

The *Arabidopsis thaliana* protein TRANSTHYRETIN-LIKE (TTL) is a potential substrate of BR-INSENSITIVE-1 (BRI1) and is involved in brassinosteroid (BR)-mediated plant growth [16]. Moreover, TTL has also been found to act as a bifunctional enzyme that catalyses two steps in the allantoin biosynthesis pathway [17, 18]. BRs, a group of polyhydroxylated steroid hormones, play important roles in the regulation of vegetative and reproductive development in addition to the response to stress [19–21]. While Allantoin serves as a vehicle for symbiotically fixed nitrogen in legume plants or nitrogen recycling and remobilization in non-legume plants [22–24]. In addition, allantoin also accumulates in plants under stress conditions [25–29], alleviating reactive oxygen species (ROS) accumulation and activating the production of abscisic acid (ABA), thereby enhancing plant abiotic stress tolerance [30–32]. Therefore, TTL represents a regulator of plant growth and may also perform important roles in stress tolerance.

Previously, we found that BYPASS1-LIKE (B1L) acts as a positive regulator in the tolerance of plants to freezing [33]. B1L interacts with 14–3-3λ, resulting in a reduction in the degradation of CBFs that improves the freezing tolerance of *Arabidopsis* [33]. B1L belongs to the DUF793 protein family, which contains at least 12 proteins, including AT1G74450 and BYPASS1. Transcriptomics analysis indicates that *AT1G74450* and *B1L* are both responsive to multiple abiotic stresses [34]. Furthermore, the overexpression of At1g74450 results in stunted plant height and reduced male fertility [35]. BYPASS1 participates in the production of a root-sourced signal that arrests shoot development via cytokinin signalling [36, 37]. Interestingly, the retarded development phenotypes of *bypass1* were more extreme under low temperature conditions than under normal or high temperature conditions [36]. Thus, DUF793 family proteins may represent potential regulators of the trade-off between stress tolerance and development.

Therefore, we investigated whether B1L participates in plant growth regulation, except for its function in the regulation of cold tolerance. In this study, we found that B1L interacts with TTL, and both of them participate not only in cold tolerance but also in the regulation of plant growth. Additionally, we found that BR and allantoin may also be involved in these processes, indicating a connection between plant growth and cold tolerance via BR or allantoin.

## Results

### TTL directly interacted with B1L

**Fig. 1:**
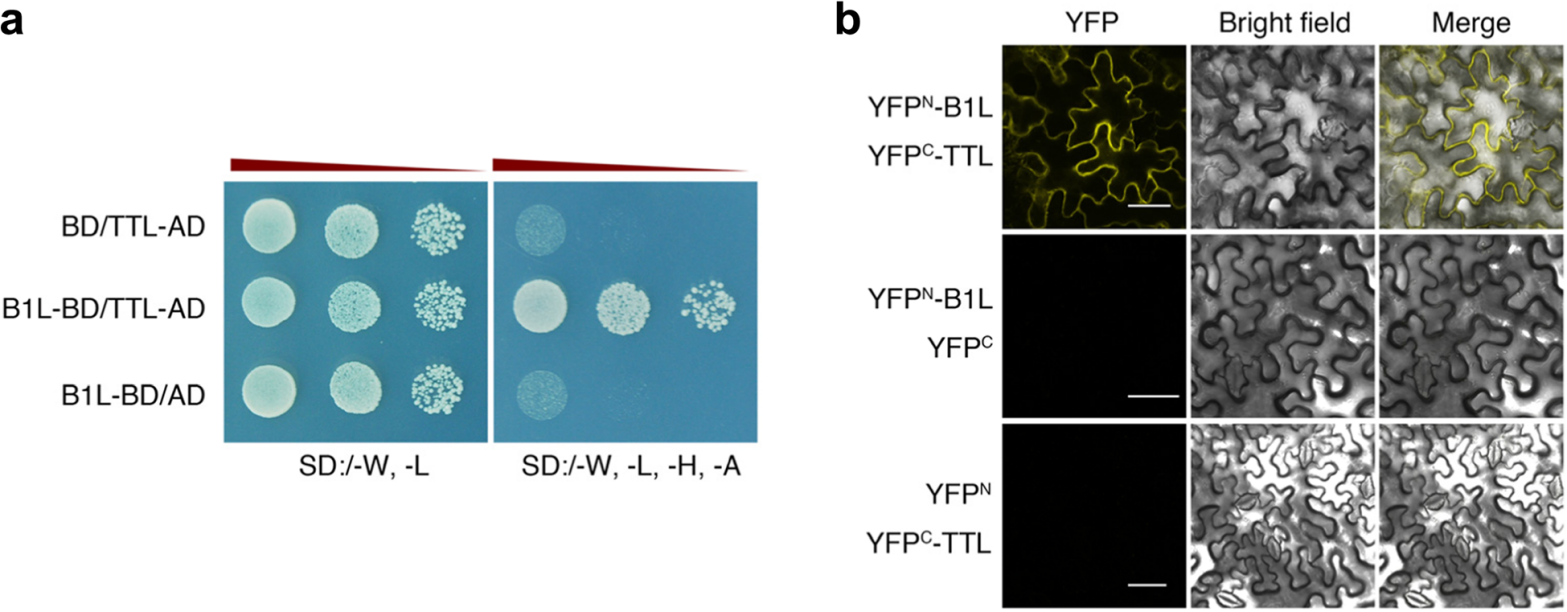
B1L interacts with TTL. **a** Y2H analysis of the interaction between B1L and TTL. Each yeast clone containing TTL-pGADT7 (TTL-AD) or pGADT7 (AD) together with B1L-pGBKT7-B1L (B1L-BD) or pGBKT7 (BD) was grown on transformation selection (SD:/−W-L) or interaction selection (SD:/−W-L-H-A) plates. Dilution of the inoculation is shown at the top of the picture. Yeast growth on SD:/−L-T-H-A indicates a positive protein-protein interaction. **b** BiFC analysis in *N. benthamiana* showing the interaction between B1L and TTL. The N-terminal half of yellow fluorescent protein (YFP^N^) was fused to B1L (B1L-YFP^N^) and the C-terminal half of yellow fluorescent protein (YFP^C^) was fused to TTL (TTL-YFP^C^). The constructs were co-transformed into tobacco leaf cells, and fluorescence images were obtained by confocal microscopy. Panels from left to right show signals of yellow fluorescence, a bright-field image, and an overlay of yellow fluorescence and bright-field image images. Bar = 50 μm

To investigate the biological function of B1L in *Arabidopsis*, we used a yeast two-hybrid (Y2H) screening system to select B1L-interacting proteins previously [33], and TTL was selected as one of the candidate proteins. In this study, Y2H and bimolecular fluorescence complementation (BiFC) assays were performed to confirm the interaction between B1L and TTL. When B1L was fused with the Gal4 DNA binding domain (BD) and TTL was fused with the Gal4 activation domain (AD) and then co-transformed into the yeast strain AH109, the Y2H assay showed that B1L interacted with TTL (Fig. 1a). Consistent with this result, the reconstituted YFP fluorescence was visualized when the BiFC assay was performed using transiently co-expressed B1L-YFP^N^ and TTL-YFP^C^ in *Nicotiana benthamiana* leaves (Fig. 1b). As a result, we found that B1L could interact with TTL in vitro and in plant cells.

### *ttl* knockout mutants exhibited enhanced seedling growth, whereas *TTL*-overexpressing lines exhibited retarded growth

**Fig. 2:**
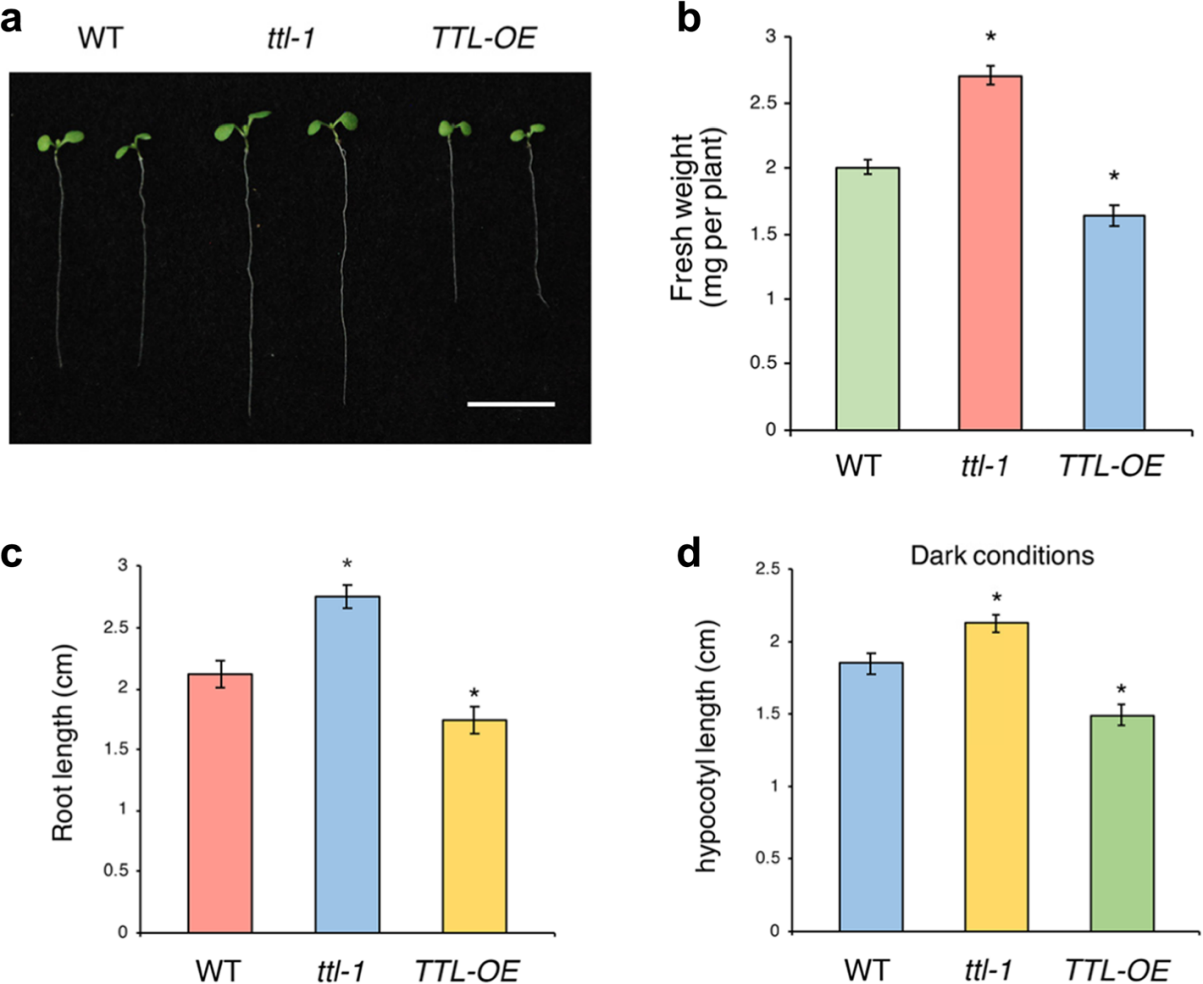
TTL restrains *Arabidopsis* seedling development. **a** Phenotypic comparison between 7-day-old *ttl-1*, TTL-OE and WT seedlings. Bar = 1 cm. **b** Fresh weight (mg) of *ttl-1*, TTL-OE, and WT seedlings showed in (**a**). **c** Primary root length of *ttl-1*, TTL-OE, and WT seedlings showed in (**a**). **d** Hypocotyl growth of 7-day-old *ttl-1*, TTL-OE, and WT seedlings in the dark conditions. All seedlings were grown on MS plates at 22 °C in a 16 h:8 h light:dark cycle (**a**, **b**, and **c**) or for 24 h in the dark (**d**). Data in (**b**, **c**, and **d**) are expressed as the mean value ± SEM (*n* = 24). Asterisks indicate significant differences (*p < 0.05) from the wild type

To investigate the biological roles of TTL, two *TTL* T-DNA insertion lines, termed *ttl-1* and *ttl-2*, were obtained from the Arabidopsis Biological Resource Center (ABRC). The *TTL* genomic sequence possesses four exons and three introns (Fig. S1a). A T-DNA insertion was located in the *ttl-1* mutant within the third intron, located 771 bp downstream of the initiation codon of TTL (Fig. S1a). In the *ttl-2* mutant, a T-DNA was inserted into the first exon, located 67 bp downstream of the initiation codon (Fig. S1a). RT-PCR with total RNAs isolated from wild type Col-0, *ttl-1*, and *ttl-2* confirmed that *TTL* was completely knocked-out in both of the two T-DNA insertion lines (Fig. S1b). One-week-old *ttl-1 and ttl-2* seedlings both displayed a promoted developmental phenotype compared with the wild type (Fig. S2a; Fig. 2a). The fresh weights of *ttl-1 and ttl-2* mutants were greater than those of the wild type (Fig. S2b; Fig. 2b). The primary roots of *ttl-1* and *ttl-2* mutant seedlings were longer than those of the wild type (Fig. S2c; Fig. 2c). The hypocotyls of *ttl-1* and *ttl-2* mutants were also longer than those of the wild type in the dark conditions (Fig. S2d; Fig. 2d), which was consistent with the findings of previous studies [16]. On the contrary, one-week-old transgenic plants overexpressing *TTL* driven by the 35S promoter (*TTL-OE*) exhibited an inhibited developmental phenotype compared with the wild type (Fig. 2a). It had a lower fresh weight and a shorter primary root (Fig. 2b, c). The hypocotyls of *TTL-OE* were also shorter than those of the wild type in the dark conditions (Fig. 2d). These results reveal that TTL negatively affects seedling growth and development.

### *b1l* knockout mutant displayed promoted seedling growth, whereas *B1L*-overexpressing lines exhibited retarded growth

**Fig. 3:**
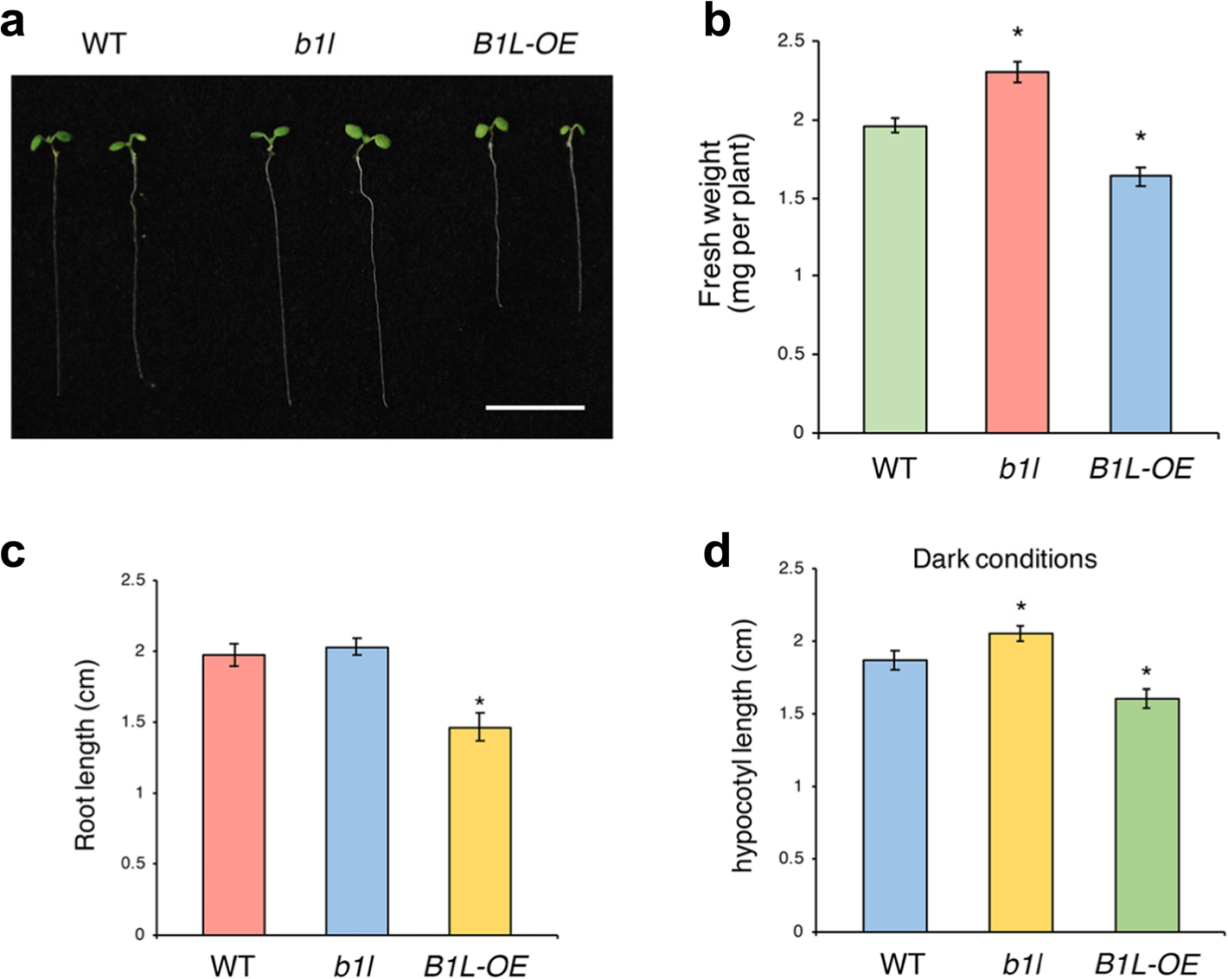
B1L inhibits *Arabidopsis* seedling development. **a** Phenotypic comparison between 7-day-old *b1l*, B1L-OE and WT seedlings. Bar = 1 cm. **b** Fresh weight (mg) of *b1l*, B1L-OE, and WT seedlings showed in (**a**). **c** Primary root length of *b1l*, B1L-OE, and WT seedlings showed in (**a**). **d** Hypocotyl growth of 7-day-old *b1l*, B1L-OE, and WT seedlings in the dark conditions. All seedlings were grown on MS plates at 22 °C in a 16 h:8 h light:dark cycle (**a**, **b**, and **c**) or for 24 h in the dark (**d**). Data in (**b**, **c**, and **d**) are expressed as the mean value ± SEM (*n* = 24). Asterisks indicate significant differences (*p < 0.05) from the wild type

A *B1L* T-DNA insertion line (*b1l*) and a *B1L*-overexpressing line (B1L-OE), which had been used in our previous study [33], were used to ascertain whether B1L also participates in these seedling growth processes. One-week-old *b1l* mutants displayed a promoted developmental phenotype compared with wild type seedlings, whereas *B1L-OE* exhibited an adverse phenotype (Fig. 3a). The fresh weight of *b1l* seedlings was greater than that of the wild type, whereas *B1L-OE* had a lower fresh weight (Fig. 3b). Although *b1l* mutants had a similar root length to the wild type, the primary root of *B1L-OE* was significantly shorter (Fig. 3c). The hypocotyls of *b1l* mutants were longer than those of wild type plants in the dark conditions, whereas those of *B1L-OE* were shorter than those of wild type plants (Fig. 3d). These results indicate that B1L, similar to TTL, negatively affects plant growth and development.

To investigate genomic interactions between B1L and TTL, we generated a *b1l ttl* double mutant by crossing *b1l* with *ttl-1*. The developmental phenotype of *b1l ttl* was similar to that of *ttl1–1* and *b1l*, which were all promoted phenotypes (Fig. S3a). The root and hypocotyl length and fresh weight of *b1l ttl* suggested highly similar growth characteristics to those of *ttl-1* (Fig. S3). These results indicate that TTL and B1L both affects seedling growth in *Arabidopsis*.

### *ttl-1* mutant was freezing-tolerance, whereas *TTL-OE* was freezing-sensitive, to cold treatment

**Fig. 4:**
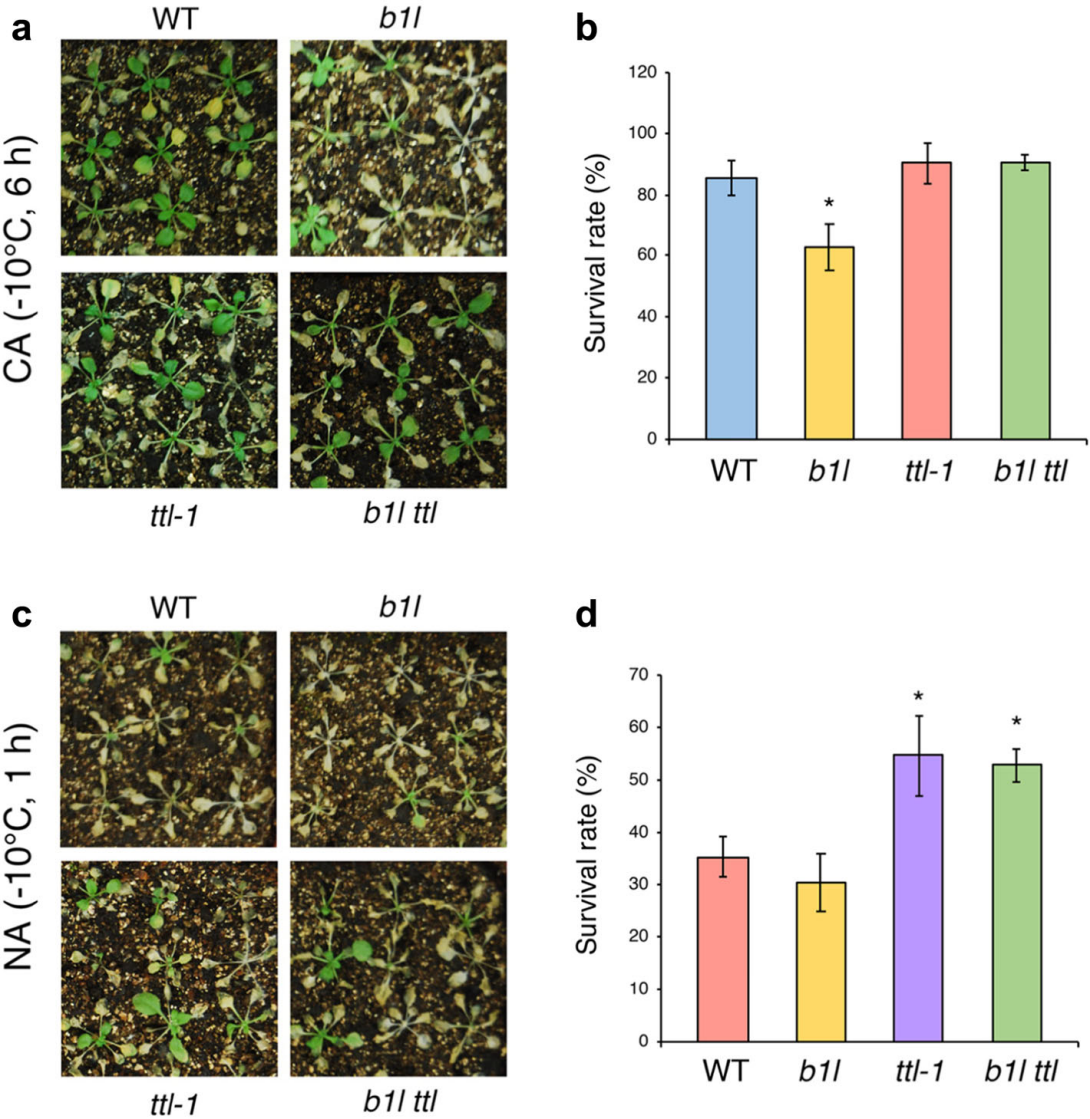
*ttl-1* and *b1l ttl* mutants were both more tolerant to freezing than WT under non-acclimation conditions. Freezing tolerance (**a**, **c**) and survival rates (**b**, **d**) of 3-week-old WT, *b1l*, *ttl-1*, and *b1l ttl* under non-acclimated (NA) or cold-acclimated (CA) conditions. Seven-day-old seedlings grown on MS plates were transplanted to soil and grown at 22 °C for 2 weeks under long day conditions (light:dark, 16 h:8 h). The plants were then treated at − 10 °C for 1 h (NA) or were pretreated at 4 °C for 3 days and then treated at − 10 °C for 6 h (CA). For each line, the survival rate assay was performed with approximately 64 plants and then scored 5 days later. The data are shown as the means of four independent biological replicates ± SEM. Asterisks indicate significant differences (*p < 0.05) from the wild type

As B1L was previously found to modulate plant freezing tolerance [33], whether TTL was able to participate in the regulation of freezing tolerance was investigated. The *b1l* mutant was more sensitive to freezing than the wild type under cold-acclimation (CA) conditions (Fig. 4a, b), as in our previous results [33], whereas the *ttl-1* mutant was more tolerant to freezing temperature than the wild type under non-acclimation (NA) conditions (Fig. 4c, d). The *b1l ttl* mutant displayed tolerance to freezing similar to that of *ttl-1* (Fig. 4). *TTL-OE* plants were also used to perform the plant freezing assay, and they were more sensitive to freezing than the wild type (Fig. S4). These results indicate that TTL negatively affects freezing tolerance in *Arabidopsis*.

### BRs provide no significant contribution to B1L-mediated seedling growth under normal conditions

**Fig. 5:**
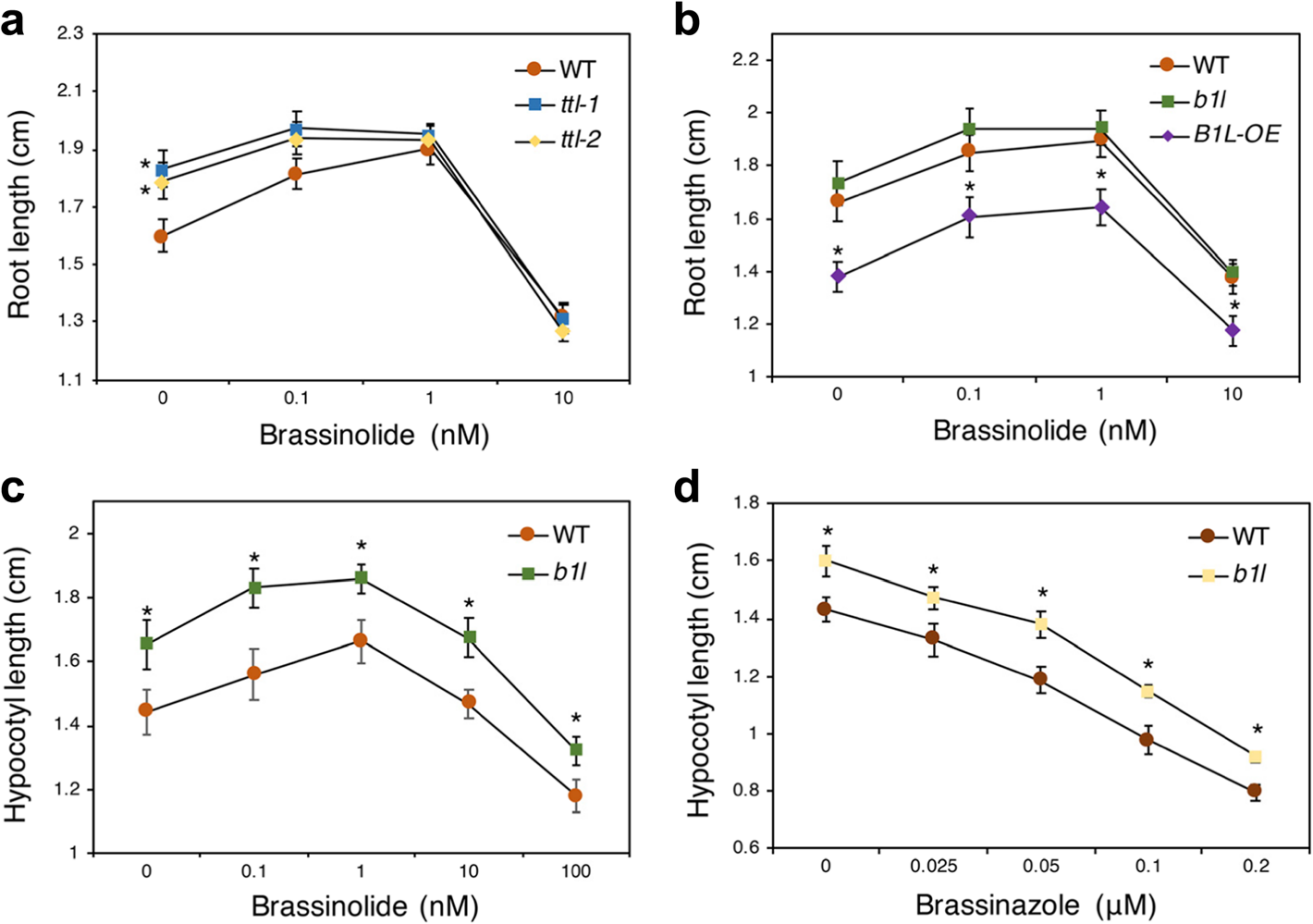
BR restrained the promoted development of the *ttl-1* mutant, but not the *b1l* mutant, under normal conditions. **a** Primary root length of 7-day-old wild type, *ttl-1*, and *ttl-2* seedlings after treatment with brassinolide, a familiar compound used to analyse the function of BRs in plant growth. **b** Primary root length of 7-day-old wild type, *b1l*, and B1L-OE seedlings after treatment with brassinolide. Seedlings in (**a**) and (**b**) were germinated and grown on MS plates containing increased concentrations of brassinolide at 22 °C in a 16 h:8 h light:dark cycle. **c** Hypocotyl growth of 7-day-old wild type and *b1l* seedlings with brassinolide treatment in the dark. The different concentrations of brassinolide (0.1, 1, 10, 100 nM groups) in panels a, b and c were all dissolved in 80% ethanol. After filter sterilization, they were added to MS plates [1:10000 (v/v)]. The MS plates with 80% ethanol [1:10000 (v/v)] were used as controls (0 nM group). **d** Hypocotyl growth of 7-day-old wild type and *b1l* seedlings with brassinazole treatment in the dark. Brassinazole is an inhibitor of BR biosynthesis. The different concentrations of brassinazole (0.025, 0.05, 0.1, 0.2 μM groups) were all dissolved in DMSO and then added to MS plates [1:1000 (v/v)]. MS plates with DMSO [1:1000 (v/v)] were used as a control (0 μM group). Seedlings in (**c**) and (**d**) were germinated and grown on MS plates containing increased concentrations of brassinolide or brassinazole at 22 °C grown in the dark condition. Each data point in panels (**a**, **b**, **c**, and **d**) represents the mean value ± SEM (*n* = 24). Asterisks indicate significant differences (*p < 0.05) compared with the wild type at each brassinolide or brassinazole concentration

It has been reported that the *TTL* knockout mutant was partially insensitive to brassinolide, a familiar compound used to analyse the function of BRs in plant growth, and brassinazole, an inhibitor of BR biosynthesis [16, 38]. Therefore, we investigated whether BRs are also involved in B1L-mediated plant growth. As shown in Fig. 5a and b, the root lengths of *ttl-1*, *ttl-2*, *b1l*, B1L-OE, and wild type were all promoted in the low concentration of brassinolide (0.1 or 1 nM) compared with the mock treatment (0 nM) group and were all inhibited in the high concentration of brassinolide (10 nM). However, statistical analysis of the root length of *ttl-1* or *ttl-2* to that of wild type plants in the same concentrations of brassinolide treatment (0.1 and 1 nM) showed that *ttl* mutants have reduced BR sensitivity (Fig. 5a), consistent with previous reports [16]. Unlike *ttl* mutants, similar statistical methods indicate that the effects of brassinolide treatment on *b1l*, B1L-OE and wild type were similar (Fig. 5b).

The hypocotyl length of *b1l*, which exhibited significant differences from that of the wild type (Fig. 3d), was also measured with different concentrations of brassinolide and brassinazole treatments to determine whether B1L regulates plant growth in a BR-dependent manner. When treated with brassinolide, the hypocotyl length of wild type and *b1l* seedlings were both enhanced at low concentrations (0.1 nM and 1 nM) compared with the mock treatment (0 nM) group but were inhibited at a high concentration (100 nM) (Fig. 5c). Meanwhile, the hypocotyl length of *b1l* was persistently longer than that of the wild type at the same concentrations of brassinolide (Fig. 5c). For brassinazole treatment, the hypocotyl lengths of wild type and *b1l* seedlings were both inhibited at different concentrations (Fig. 5d), and the hypocotyl length of *b1l* was also persistently longer than that of the wild type at each concentration of brassinazole (Fig. 5d). These results further indicate that BR may not play special roles in B1L-mediated seedling growth.

### *b1l* mutants were partially insensitive to BR treatment under cold conditions

**Fig. 6:**
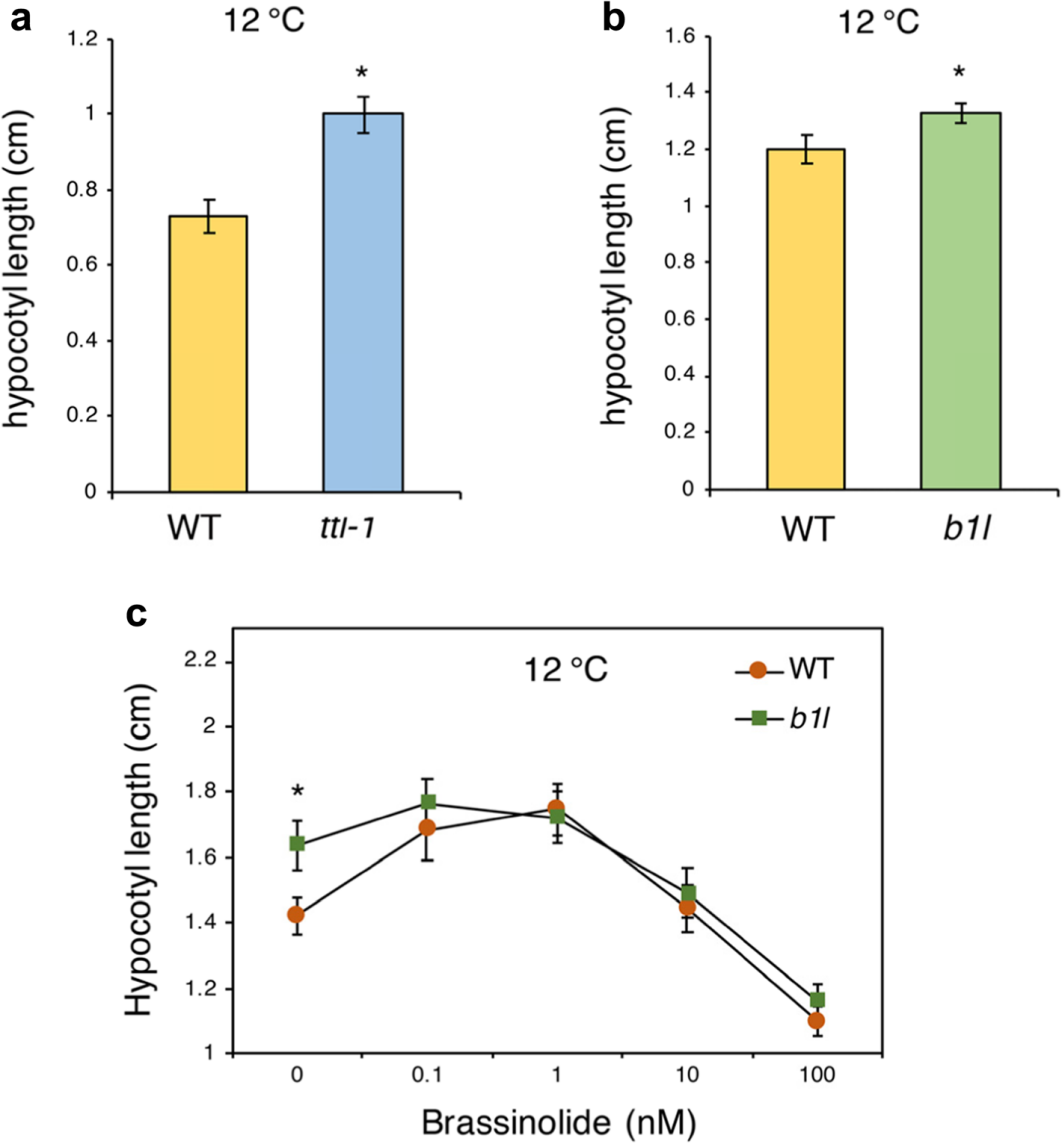
*ttl1–1* and *b1l* exhibited promoted seedling development under cold conditions, and BR restrained the promoted hypocotyl length of *b1l* mutants under cold conditions. **a** Hypocotyl growth of 2-week-old wild type and *ttl-1* seedlings under cold conditions in the dark. **b** Hypocotyl growth of 2-week-old wild type and *ttl-1* seedlings under cold conditions in the dark. Seedlings in (**a**) and (**b**) were germinated and grown on MS plates at 12 °C for 24 h in the dark. Each data point is expressed as the mean value ± SEM (*n* = 24). Asterisks indicate significant differences (*p < 0.05) compared with the wild type. **c** Hypocotyl growth of 2-week-old wild type and *b1l* seedlings with BR treatment in the dark under cold conditions. Seedlings were germinated and grown on MS plates containing increased concentrations of brassinazole at 12 °C in the dark conditions. Each data point represents the mean value ± SEM (*n* = 24). Asterisks indicate significant differences (*p < 0.05) compared with the wild type at each brassinolide concentration

As B1L and TTL both participate in the regulation of plant growth and cold tolerance, we further explored whether low temperature affects B1L- or TTL-mediated seedling growth. However, we found that the hypocotyl lengths of *ttl1–1* and *b1l* were still longer than those of the wild type under cold conditions (12 °C) (Fig. 6a, b), as in normal conditions (Figs. 2d and 3d). Intriguingly, statistical analysis of the hypocotyl elongation in *b1l* to that of wild type in the same low concentrations of brassinolide treatment (0.1 and 1 nM) showed that *b1l* mutants were less sensitive to BR treatment than wild type under cold conditions (12 °C) (Fig. 6c), despite not being under normal conditions (Fig. 5c), indicating that BR may serve special roles in B1L-mediated seedling development under cold conditions in *Arabidopsis*.

### Synthesis of allantoin was significantly inhibited in the *b1l* mutant

**Fig. 7:**
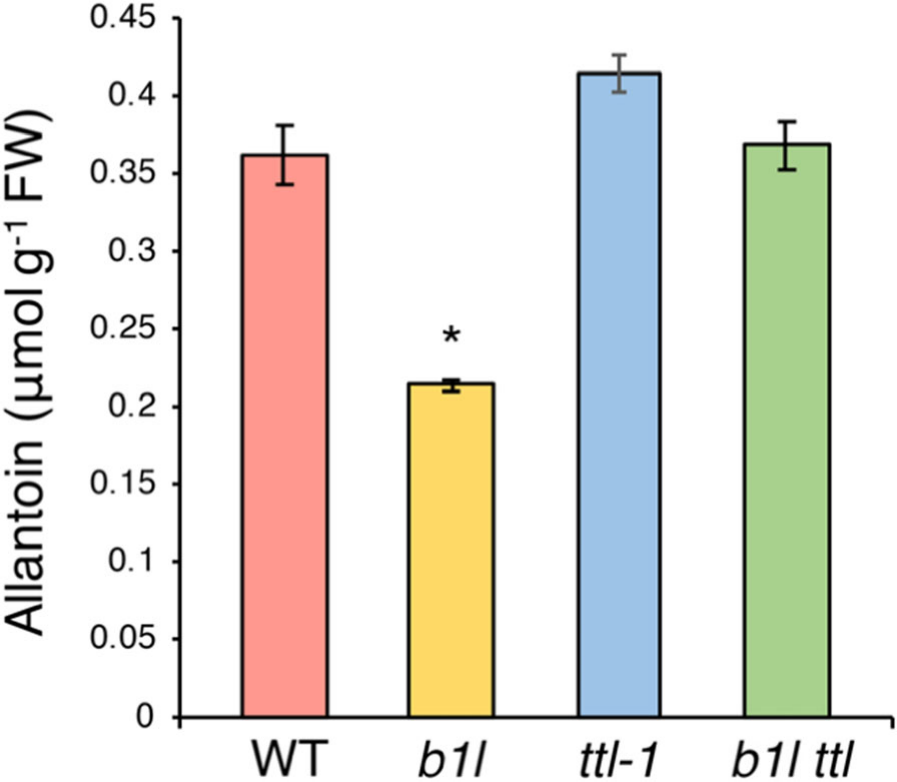
Endogenous allantoin levels in 2-week-old wild type, *b1l*, *ttl-1*, and *b1l ttl* seedlings. Data represent the means ± SEM from three independent experiments. Asterisks indicate significant differences (*p < 0.05) from the wild type

TTL has been shown to act as a bifunctional enzyme in the synthesis of S-allantoin [17, 18]. Therefore, we investigated whether *TTL* or *B1L* knockout impacted the synthesis of allantoin. The 2-week-old *b1l* mutant contained a significantly smaller quantity of allantoin than Col-0, whereas allantoin levels in *ttl-1* and *b1l ttl-1* were similar to that of the wild type (Fig. 7). This result suggests that *TTL* may represent genetic redundancy in the synthesis of allantoin, and that B1L may also be involved in the modulation of allantoin production.

## Discussion

Plants have evolved complex systems to respond to and optimize cold temperatures. Some cold stress-related genes, such as *ICE1*, *CBFs*, and *COLD-REGULATED* (*COR*) genes, have been shown to moderate plant growth. For instance, overexpression of *CBF1* restrained plant growth, at least partly through the accumulation of DELLA proteins [8, 13]. *COR27* and *COR28* negatively regulate freezing tolerance but positively regulate flowering in *Arabidopsis* [39]. ICE1 plays critical roles in diverse developmental processes, including primary seed dormancy [15], endosperm development [10], leaf and anther stomata development [9, 12, 14], and flowering [11]. In this study, we found that TTL and B1L not only participated in the regulation of cold tolerance but also in the development of seedlings.

Previously, we found that B1L interacts with 14–3-3λ to prevent the degradation of CBF proteins, thereby increasing the expression of *COR* genes to improve the freezing tolerance of *Arabidopsis* [33]. Although the *b1l 14–3-3kλ* mutant was more freezing tolerance than the wild type, the freezing tolerance of *b1l 14–3-3kλ* was less than that of *14–3-3kλ* [33], suggesting that other proteins may also be involved in B1L-mediated freezing tolerance. In this study, we found that TTL interacted with B1L (Fig. 1) and that TTL was important for B1L function in plant development and cold tolerance (Fig. S3 and Fig. 4). TTL has been showed to affect BR-mediated plant growth and catalyse allantoin biosynthesis in previous studies [16–18], we therefore analysed the effect of BR on B1L- and TTL-mediated plant growth under both normal conditions and low temperature, and also measured the concentration of allantoin in *b1l*, *ttl* and *b1l ttl* mutants. Our results reveal that BR and allantoin may also be involved to these processes. As TTL can catalyse the biosynthesis of allantoin, allantoin may serve a pivotal role in the downstream regulation of cold tolerance and development in *Arabidopsis*. The role of allantoin needs to be further elucidated in the future.

To date, extensive studies have shown that BRs play critical roles in modulating plant growth and development. In recent years, BR was also reported to increase plant tolerance to adverse environments, such as salt, drought, and cold temperatures [20, 40–42]. The transcription factors BRASSINAZOLE RESISTANT 1, BRI1 EMS SUPPRESSOR 1, and CESTA, all well characterized as being BR-controlled [26–28], can enhance freezing tolerance through both CBF-dependent and CBF-independent pathways [43, 44]. Consistent with this finding, the GSK3-like kinase BIN2, which phosphorylates these transcription factors to promote their degradation in the absence of BR [45, 46], exhibits negative roles in regulating the freezing tolerance of *Arabidopsis* [44]. Studies in UV-B, drought, and pathogen stresses indicate that BR may function as a cross-talk between growth and stress responses [47, 48]. We found that brassinolide serves different roles in hypocotyl elongation in *b1l* under normal conditions and cold conditions (12 °C) (Figs. 5c and 6c), suggesting a possible connection between plant growth and cold tolerance via BR.

Allantoin has also been shown to activate stress-related gene expression and the production of ABA, thereby alleviating ROS accumulation and cell death [30–32]. We found that *b1l* contained a smaller quantity of allantoin than the wild type (Fig. 7). Consistent with this result, *b1l* was freezing sensitive compared with the wild type (Fig. 4). However, a new question arises: As recombinant TTL could catalyse two enzymatic reactions to produce allantoin in vitro [17, 18], why did the *ttl-1* mutant did not show significantly different quantities of allantoin compared with the wild type (Fig. 7)? We hypothesize that the *TTL* gene may be genetically redundant in *Arabidopsis*. Our results indicate that allantoin may participate in B1L-mediated plant growth and cold tolerance.

## Conclusion

B1L interacts with TTL, and both participate in the regulation of development and cold tolerance in *Arabidopsis*. BR and allantoin may also participate in these processes through B1L and TTL. As BR and allantoin can be exogenously applied to crops, it is meaningful and necessary to determine their roles in balancing plant growth and cold tolerance.

## Methods

### Plant materials

All mutants and transgenic lines used in this study were created from the Columbia (Col-0) wild type strain. *Ttl-1* (SALK_137289) and *ttl-2* (CS_875458) were obtained from ABRC (Arabidopsis Biological Resource Center). *b1l* (SALK_019913) was obtained from Arabidopsis Biological Resource Center. *B1L-OE* was generated in our lab. *b1l* and *B1L-OE* have been used in a previous study of ours [33]. *b1l ttl* was generated by crossing *b1l* and *ttl-1.* TTL-overexpressing transgenic lines (*TTL-OE*) were obtained by amplifying the TTL-coding sequence and cloning the resulting PCR product into the pEarlygate104 Gateway binary vector. The T-DNA insertion mutant lines and overexpression lines used in this study are all homozygous plants.

All primer sequences used in this study are listed in Table S1.

### Measurement of fresh weight and lengths of primary root and hypocotyl

Seedlings were grown on vertical MS agar plates in order to measure the fresh weight and length of the primary root and hypocotyl. After sowing under long day conditions at room temperature (22 °C), the primary root length of the seedlings was measured, the fresh weight was quantified, and the seedlings were photographed after at 7 days. Following sowing in the dark at both room temperature (22 °C) and under cold conditions (12 °C), the length of the seedling hypocotyls was measured after 7 days and 2 weeks, respectively.

For treatment with BR and BR inhibitors, the seedlings were grown on MS plates containing different concentrations of brassinolide (0, 0.1, 1, 10, 100 nM) or brassinazole (0, 0.025, 0.05, 0.1, 0.2 μM). Brassinolide is a familiar compound used to analyse the function of BRs in plant growth, and brassinazole is an inhibitor of BR biosynthesis. Root length and hypocotyl length data were obtained from these experiments.

### Plant freezing assay

The plant freezing assay was performed as previously described [33]. Briefly, 64 plants of each strain grown for 3 weeks under long-day conditions were used to conduct the assay. The plants were alternately placed in a controlled-temperature chamber (MIR-254; SANYO) at 0 °C, and the temperature then decreased by 1 °C/h. After treatment at the selected temperature, the plants were maintained at 4 °C for 12 h and then at 22 °C for 5 days to recover to ascertain the survival rate. The experiments were repeated 3 times for statistical analysis.

### RT-PCR assay

Total RNA from 2-week-old seedlings was extracted using an RNAprep Pure Plant Kit (TIANGEN). The RNA was then reverse transcribed to cDNA using a RevertAid First Strand cDNA Synthesis kit (Thermo Scientific). RT-PCR was performed using the gene-specific primers shown in Table S1. The number of cycles was 28 for amplifying *TTL* and *ACTIN2*/*8*. The PCR products were detected by electrophoresis in 1.5% agarose gels and then stained with ethidium bromide.

### Measurement of allantoin

Allantoin was measured in two-week-old seedlings grown on MS plates. The allantoin in each sample was converted to glyoxylate and then measured using a colorimetric method, as previously described [49, 50].

### Y2H and BiFC assays

For the Y2H assay, the full-length cDNA of B1L and TTL was PCR-amplified and cloned into the pDONR vector and subcloned into pGBKT7-GW and pGADT7-GW respectively. B1L fused with the DNA-binding domain of GAL4 in the yeast vector pGBKT7-GW (B1L-BD) or pGBKT7 (BD) together with TTL fused with the activation domain of GAL4 in the yeast vector pGADT7-GW (TTL-AD) or pGADT7 (AD) was transformed into the yeast strain AH109. The yeast transformants were screened on synthetic dextrose minimal medium (SD) lacking leucine and tryptophan (SD/: -W, −L). The resulting yeast cells were then transplanted on (SD/: -W, −L) and SD lacking leucine, tryptophan, adenine, and histidine (SD/: -W, −L, −H, −A), respectively. The pictures were taken 5 days later.

For the BiFC assay, B1L- and TTL-coding sequences were amplified and cloned into pNYFP-X and pCCFP-X Gateway binary vectors, respectively. B1L fused with the N-terminal of YFP (amino acids 1–172) in the vector pNYFP-X (YPF^N^-B1L), pNYFP-X (YPF^N^), TTL fused with C-terminal of YFP (amino acids 173–238) in the vector pCCFP-X (YPF^C^-TTL), and pCCFP-X (YPF^C^) were introduced into GV3101, respectively. Then, YPF^N^-B1L and YPF^C^-TTL, YPF^N^ -B1L and YPF^C^, or YPF^N^-B1L and YPF^C^-TTL were co-transformed into *N. benthamiana* leaves. The YFP fluorescence signal was measured after 2 days using a confocal microscope (Leica SP8).

## Supplementary information

**Additional file 1: Figure S1.** Description of two *TTL* T-DNA insertion mutants. **Figure S2.** Loss-of-function of TTL results in a promoted seedling development. **Figure S3.***b1l ttl* mutants result in promoted seedling development similar to *ttl-1*. **Figure S4.** TTL-overexpressing line was more freezing sensitive than WT. **Table S1.** Oligonucleotide sequences of the primers used in this study.

## Data Availability

All data generated or analysed during this study are included in this published article and its supplementary information files. The datasets used and/or analysed during the current study are available from the corresponding author on reasonable request. The plant materials used in this article can be accessed from the corresponding author on reasonable request.

## Abbreviations

B1L: BYPASS1-LIKE
TTL: TRANSTHYRETIN-LIKE
BR: Brassinosteroid
BRI1: BR-INSENSITIVE-1
Y2H: Yeast two-hybrid
BiFC: Bimolecular fluorescence complementation
CA: Cold-acclimation
NA: Non-acclimation
WT: Wild type
