# Supplementary material for: TRANSTHYRETIN-LIKE and BYPASS1-LIKE co-regulate growth and cold tolerance in Arabidopsis
Source: BMC Plant Biol. 2020 Jul 14;20:332. doi: 10.1186/s12870-020-02534-w (PMC7362626; doi:10.1186/s12870-020-02534-w)
Supplement: Supplementary file 1 — Additional file 1: Figure S1. Description of two TTL T-DNA insertion mutants. Figure S2. Loss-of-function of TTL results in a promoted seedling development. Figure S3.b1l ttl mutants result in promoted seedling development similar to ttl-1. Figure S4. TTL-overexpressing line was more freezing sensitive than WT. Table S1. Oligonucleotide sequences of the primers used in this study. [file 12870_2020_2534_MOESM1_ESM.docx]

**SUPPLEMENTAL MATERIALS**


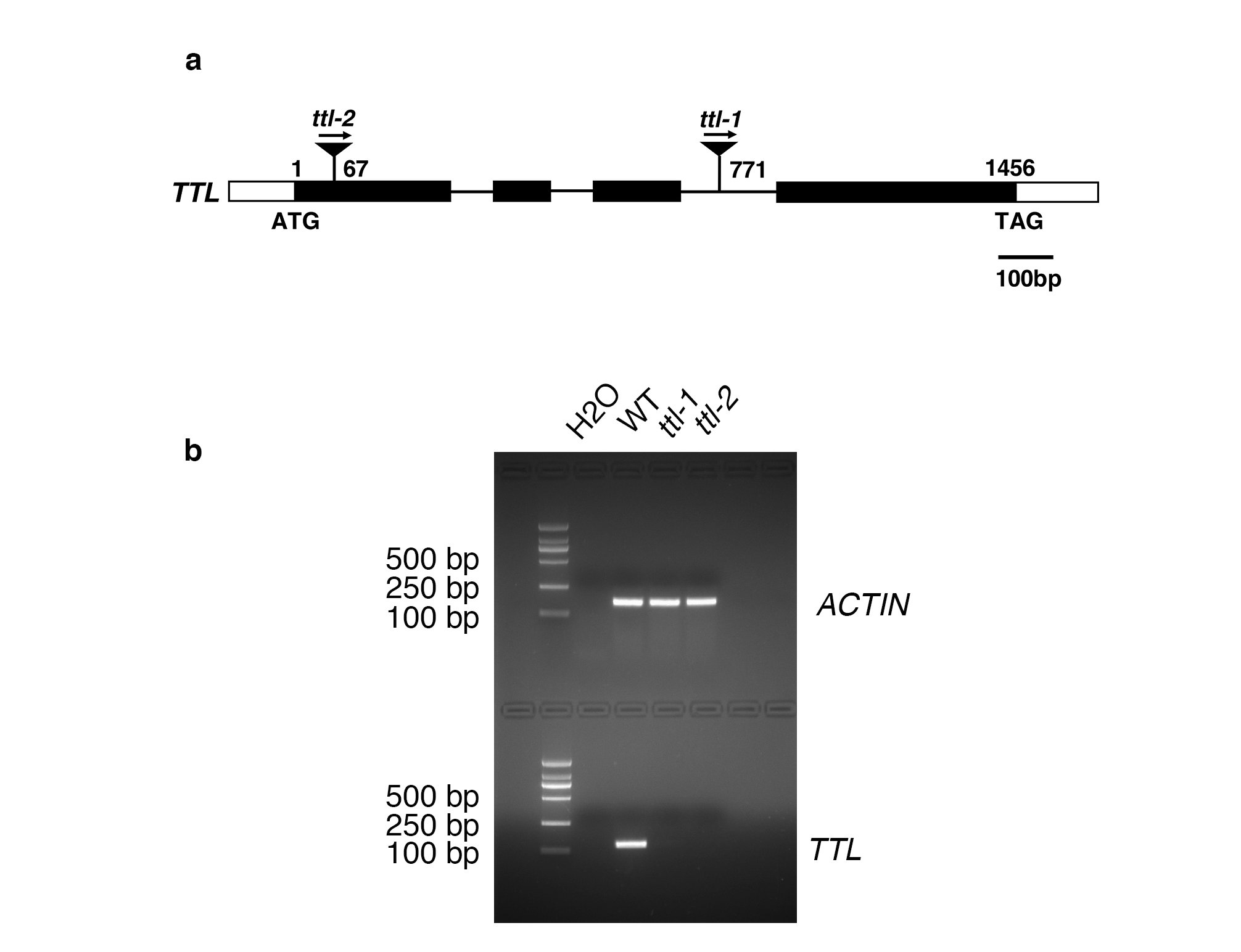


**Figure S1.** Description of two *TTL* T-DNA insertion mutants. (**a**) Schematic of the T-DNA insertion mutants *ttl-1* and *ttl-2*. White frames indicate 5′-or 3′-untranslated regions (UTRs), black frames indicate exons, lines indicate introns, and black triangles indicate the position of the T-DNA insertion mutant. (**b**) Expression of *TTL* gene in 12-day-old *ttl-1* and *ttl-2* mutants as detected by RT-PCR analysis. RT-PCR was performed using *TTL* specific primers. *ACTIN2*/*8* was used as a control


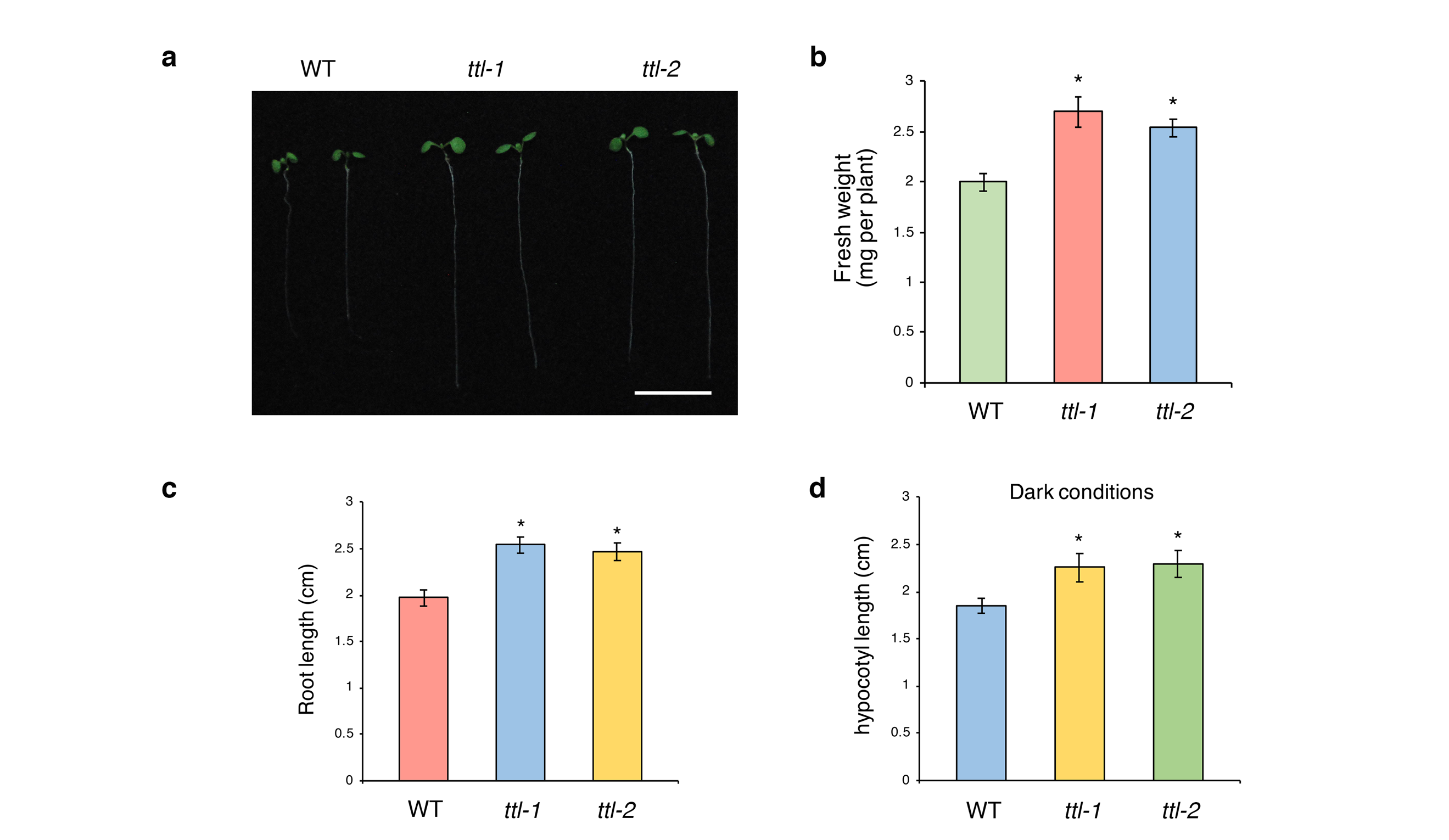


**Figure S2.** Loss-of-function of TTL results in a promoted seedling development. (**a**) Phenotypic comparison between 7-day-old *ttl-1*, *ttl-2* and WT seedlings. Bar = 1 cm. (**b**) Fresh weight (mg) of *ttl-1*, *ttl-2*, and WT seedlings showed in (a). (**c**) Primary root length of *ttl-1*, *ttl-2*, and WT seedlings showed in (a). (**d**) Hypocotyl growth of 7-day-old *ttl-1*, *ttl-2* and WT seedlings in the dark conditions. All seedlings were grown on MS plates at 22°C in a 16 h:8 h light:dark cycle (a, b, and c) or for 24 h in the dark (d). Data in (b, c, and d) are expressed as the mean value ± SEM (n = 24). Asterisks indicate significant differences (*p < 0.05) from the wild type.


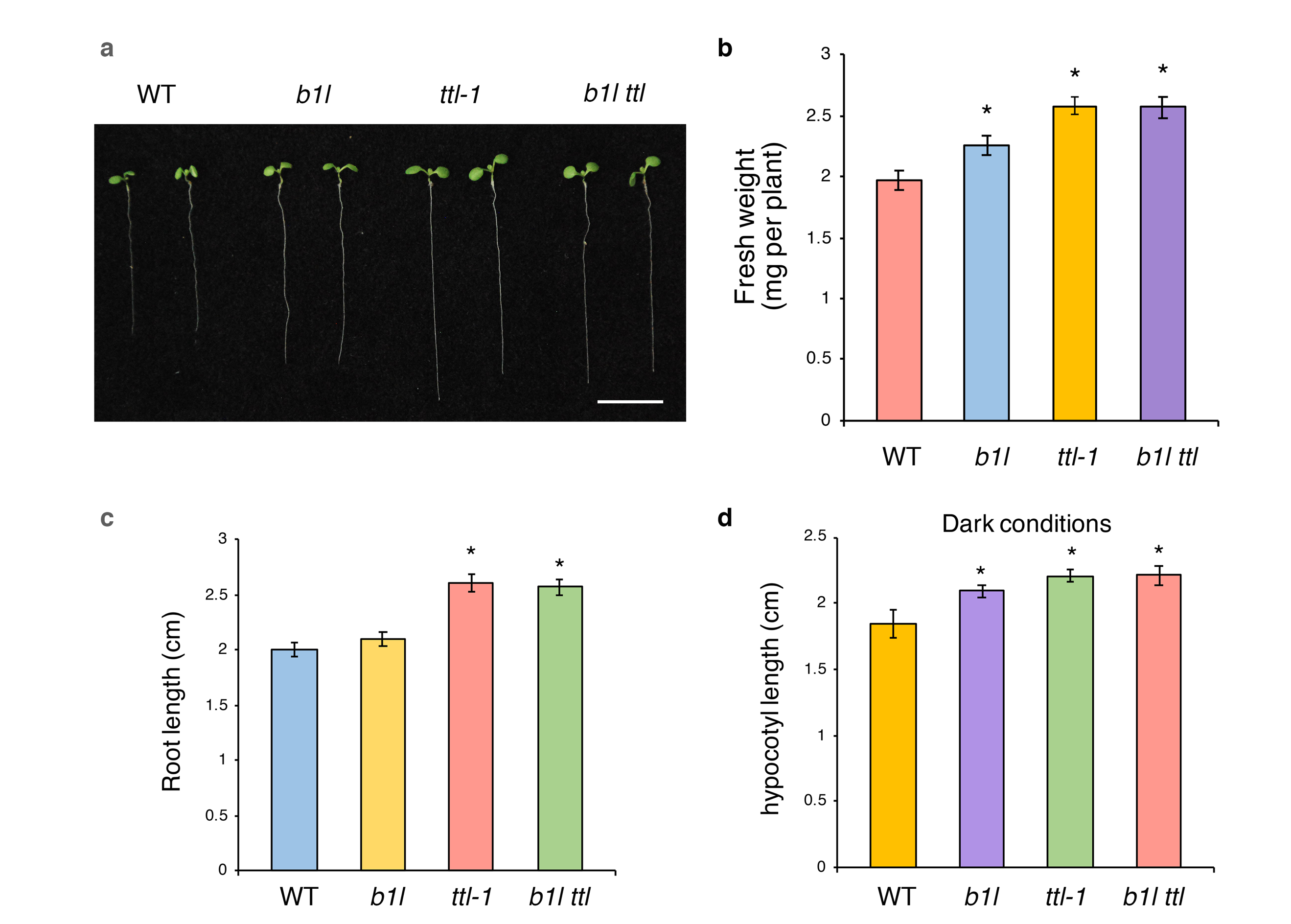


**Figure S3.** *b1l ttl* mutants result in promoted seedling development similar to *ttl-1*. (**a**) Phenotypic comparison between 7-day-old WT, *b1l*, *ttl-1*, and *b1l ttl* seedlings. Bar = 1 cm. (**b**) Fresh weight (mg) of WT, *b1l*, *ttl-1*, and *b1l ttl* seedlings showed in (a). (**c**) Primary root length of *b1l*, *ttl-1*, *b1l ttl*, and WT seedlings showed in (a). (**d**) Hypocotyl growth of 7-day-old *b1l*, *ttl-1*, *b1l ttl*, and WT seedlings in the dark conditions. All seedlings were grown on MS plates at 22°C in a 16 h:8 h light:dark cycle (a, b, and c) or for 24 h in the dark (d). Data in (b, c, and d) are expressed as the mean value ± SEM (n = 24). Asterisks indicate significant differences (*p < 0.05) compared with the wild type.

.


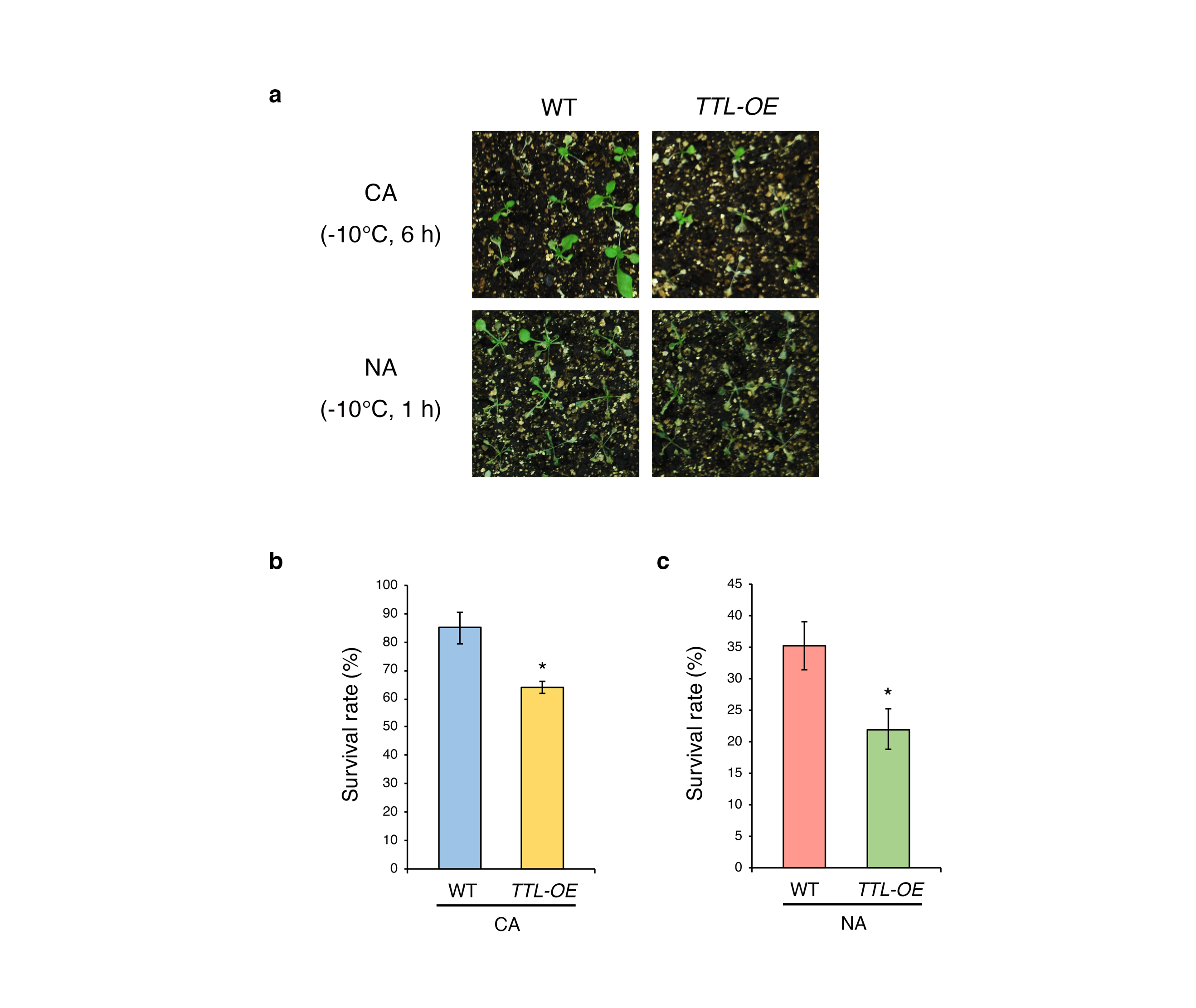


**Figure S4.** TTL-overexpressing line was more freezing sensitive than WT. Freezing tolerance (A) and survival rates (B) of 3-week-old wild type Col-0 and TTL-OE under non-acclimated (NA) or cold-acclimated (CA) conditions. Seven-day-old seedlings grown on MS plates were transplanted to soil and grown at 22°C for 2-week. Then, the plants were treated at −10°C for 1 h (NA) or were pretreated at 4°C for 3 days and then treated at −10°C for 6 h (CA). For each line, the survival rate assay was performed with about 64 plants and scored 5 days later. The data are shown as means of three independent biological replicates ± SD. Asterisks indicate significant differences (*p < 0.05) from wild type.

**Table S1** Oligonucleotide sequences of the primers used in this study

| Primers used for Mutant genotyping | |
| --- | --- |
| Name | Sequence 5'-3' |
| *ttl-1*-LP | ATGAATGGAAGGTTTGCTGTG |
| *ttl-1*-RP | TGGGGAATACTTTGCAGTGTC |
| LBb1.3 | ATTTTGCCGATTTCGGAAC |
| *ttl-1*-LP | AGCGGTCATCATCGTACTGAC |
| *ttl-1*-RP | TTGGAAATGCAAAAGTTGACC |
| LB2 | GCTTCCTATTATATCTTCCCAAATTACCAATACA |
| Primers used for *TTL* plasmids constructing | |
| Name | Sequence 5'-3' |
| *TTL*-CDS-F | ATGGCGATGGAGATCGGAGAAG |
| *TTL*-CDS-R | GCTCCCACGGTATGTGGAG |
| Primers used for RT-PCR | |
| Name | Sequence 5'-3' |
| *ACTIN2/8*-F | GGTAACATTGTGCTCAGTGGTGG |
| *ACTIN2/8*-R | AACGACCTTAATCTTCATGCTGC |
